# Potassium Efflux and Cytosol Acidification as Primary Anoxia-Induced Events in Wheat and Rice Seedlings

**DOI:** 10.3390/plants9091216

**Published:** 2020-09-16

**Authors:** Vladislav V. Yemelyanov, Tamara V. Chirkova, Maria F. Shishova, Sylvia M. Lindberg

**Affiliations:** 1 Department of Genetics and Biotechnology, Saint-Petersburg State University, Universitetskaya em., 7/9, 199034 Saint-Petersburg, Russia; 2 Department of Plant Physiology and Biochemistry, Saint-Petersburg State University, Universitetskaya em., 7/9, 199034 Saint-Petersburg, Russia; mim39@mail.ru (T.V.C.); mshishova@mail.ru (M.F.S.); 3 Department of Ecology, Environment and Plant Sciences, Stockholm University, SE-106 91 Stockholm, Sweden; sylvia.lindberg@su.se

**Keywords:** anoxic signaling, potassium, pH, acidification, fluorescence microscopy, *Triticum aestivum*, *Oryza sativa*

## Abstract

Both ion fluxes and changes of cytosolic pH take an active part in the signal transduction of different environmental stimuli. Here we studied the anoxia-induced alteration of cytosolic K^+^ concentration, [K^+^]_cyt_, and cytosolic pH, pH_cyt_, in rice and wheat, plants with different tolerances to hypoxia. The [K^+^]_cyt_ and pH_cyt_ were measured by fluorescence microscopy in single leaf mesophyll protoplasts loaded with the fluorescent potassium-binding dye PBFI-AM and the pH-sensitive probe BCECF-AM, respectively. Anoxic treatment caused an efflux of K^+^ from protoplasts of both plants after a lag-period of 300–450 s. The [K^+^]_cyt_ decrease was blocked by tetraethylammonium (1 mM, 30 min pre-treatment) suggesting the involvement of plasma membrane voltage-gated K^+^ channels. The protoplasts of rice (a hypoxia-tolerant plant) reacted upon anoxia with a higher amplitude of the [K^+^]_cyt_ drop. There was a simultaneous anoxia-dependent cytosolic acidification of protoplasts of both plants. The decrease of pH_cyt_ was slower in wheat (a hypoxia-sensitive plant) while in rice protoplasts it was rapid and partially reversible. Ion fluxes between the roots of intact seedlings and nutrient solutions were monitored by ion-selective electrodes and revealed significant anoxia-induced acidification and potassium leakage that were inhibited by tetraethylammonium. The K^+^ efflux from rice was more distinct and reversible upon reoxygenation when compared with wheat seedlings.

## 1. Introduction

A wide range of signal perception and transduction systems in plant cells is responsible for distinguishing and triggering a correct adaptive response [1,2]. Signal transduction pathways that are specific for different stressors include several steps that are shown to be rather universal. One of them is a cytosolic Ca^2+^ elevation at internal and external signals application [3,4,5]. Plant cells carefully maintain a low Ca^2+^ concentration in the cytosol and a significant gradient between the cell wall and a number of organelles. This balance is actively regulated by a variety of membrane transport systems (recently reviewed in [6]). Another well-documented primary signaling event is the accumulation of reactive oxygen species (ROS) [7,8,9,10]. The appearance of ROS caused by different stressors triggers a wide spectrum of reactions and is quickly eliminated by the antioxidant machinery. Both the second messengers (Ca^2+^ and ROS) are closely linked to each other and are involved in the transduction of different environmental stressors and internal signals such as phytohormones, regulatory proteins, RNAs and metabolites [6,7,11]. One important mechanism is the Ca^2+^-induced activation of NADPH-oxidase activity via its integration in sterol-rich lipid rafts [12]. On the other hand, ROS activates Ca^2+^ channels in the plasma membrane [3] and Ca^2+^-ATPases, which interfere with Ca^2+^ homeostasis [13]. Initially, weak signals of both Ca^2+^ and ROS were hypothesized to be amplified through the so-called ROS-Ca^2+^ hub [6]. The change of Ca^2+^ and ROS to an inactive state is also a necessity and supposed to be highly regulated. The cytosol Ca^2+^ elevation triggered by a variety of internal and external signals coincides with cytosolic acidification (reviewed in [14,15]). It was questionable for some time if protons had a signaling role, probably because of its involvement in metabolism. It is well known that Ca^2+^ elevation in the cytosol affects the pH level. The duration and intensity of Ca^2+^ increases might vary and thus determine the specificity of response to diverse signals (stressors, hormones, light, etc.). Different systems of H^+^ transport through the plasma membrane and tonoplast are involved. Proton pumps are supposed to be regulated on transcriptional and posttranslational levels, which make the H^+^ signature highly specific.

Electrolyte leakage is another process accompanying transient Ca^2+^, ROS and proton increases under stress conditions. A number of experimental data reveals that electrolyte leakage is mainly defined as K^+^ efflux from plant cells [16,17].

Potassium is essential for plant cells/organisms in many aspects. It is a well-known macro-nutrient. Deficiency of K^+^ results in growth arrest especially in seedlings and young organs. This ion is important for plant metabolism due to its facility to activate more than 70 enzymes [17,18]. Besides that, K^+^ serves as a charge-balancing ion, which plays an important role in the transport through the plasma membrane under the limitation of ATP and the depolarization of the membrane potential. The K^+^ gradient maintains turgor and serves as a source of energy to stimulate sucrose loading into the phloem [19,20]. Accumulated evidence indicates that the efflux depends on the type and the intensity of the stressor as well as on affected plant species and tissue. The cytosolic K^+^ concentration in plant cells is about 70–200 mM [21]. Several transporters have been shown to be involved in K^+^ accumulation: the High Affinity K^+^ transporter (HAK)–K^+^ uniporter and the Arabidopsis K^+^ Transport system 1 (AKT1)–K^+^/H^+^ symporter [18,22]. These processes require energy and depend on the external K^+^ concentration and the K^+^ vacuolar pool. The priority role in stress-induced K^+^ leakage is given to another system: Gated Outward Rectifying K^+^ efflux (GORK) channels [23]. By activation, it decreases the cytosolic potassium concentration to 10–30 mM [21]. The activity of these channels is sensitive to membrane potential depolarization through a clustering mechanism [24]. A number of experimental data combined with a bioinformatics approach suggest a possible ligand regulation of K^+^ flux through GORK channels [25]. Cyclic nucleotides (CNs), gamma-aminobutyric acid (GABA), G proteins, protein phosphatases, inositol, ROS and ATP are on a list of potential GORK regulating ligands. The presented data even stronger introduced both K^+^ and GORK in signaling cascades triggered by stress factors and led to the conclusion that potassium fulfills the role of a second messenger [17,25]. A signaling role of potassium is well documented for salt stress [18].

Oxygen deficiency is another stress factor that affects K^+^ efflux and causes severe damage to plant organisms [26,27,28,29,30]. Surprisingly, plants known as strict aerobic organisms might be affected not only by an external lack of oxygen. Different tissues or even groups of cells experience hypoxia conditions during normal plant development [31]. Thus, it becomes even more important to discover the steps of early hypoxia signal transduction. The mechanism of oxygen sensing in plants and animals are strictly diverse. However, low oxygen regulates the function of various K^+^ channels in mammals. Recently, plant cell ion channels have been identified as potential candidates for low oxygen sensing in flooded roots [32]. However, additional studies are required to estimate a possible modulation of K^+^ transport through an ERF-VII-mediated response to a lack of oxygen. Nevertheless, hypoxic/anoxic environment causes K^+^ efflux from plant cells and GORK channels are supposed to play a crucial role in this process [25]. Under hypoxic stress, cytosolic K^+^ participates in the regulation of several physiological processes possibly including the formation of aerenchyma [33].

Oxygen deprivation triggers Ca^2+^ signaling. The alteration in [Ca^2+^]_cyt_ is a fast and intensive reaction for limitation in oxygen supply and energy deficiency [34,35,36,37]. Ca^2+^ elevation in cytosol coincides with acidification [15]. A probable reason is the hypoxia-induced inhibition of the plasma membrane H^+^-ATPase and tonoplast H^+^-ATPase activities due to the lack of ATP.

Signaling via K^+^ efflux is transient and therefore the timing of this event should be evaluated. It was found that plant species and even plant organs differ in their tolerance to hypoxia. Some data revealed that the intensity of K^+^ efflux corresponded to plant sensitivity to oxygen deprivation and depended on metabolic activity [30]. It would be of interest to integrate [K^+^]_cyt_ into the schedule of other intracellular signaling events such as Ca^2+^ elevation and acidification under a lack of oxygen. Earlier we provided a comparison of calcium signaling during anoxia in two well-known agricultural plants such as rice and wheat, which differ in tolerance to oxygen limitation [35]. Rice cells were shown to be more reactive to oxygen depletion and depended on both external and internal Ca^2+^ stores. This study focuses on a possible alteration of intracellular potassium concentration, [K^+^]_cyt_, and cytosolic pH, pH_cyt_, during anoxic signal transduction in the protoplasts from wheat and rice.

The mentioned ions have not only a signaling role but also are very important in the regulation of metabolic processes including those at stress conditions. We therefore also estimated the cell-level events at a whole plant level. We investigated the ion changes after reoxygenation as well. It is of special interest how proton and Ca^2+^ accumulation inside cells and active potassium efflux would reflect processes during long-time stress applications and regulate the ion exchange with an external medium.

## 2. Results

### 2.1. Influence of Oxygen Deprivation on [K^+^]_cyt_ in Wheat and Rice Leaf Protoplasts

**Figure 1:**
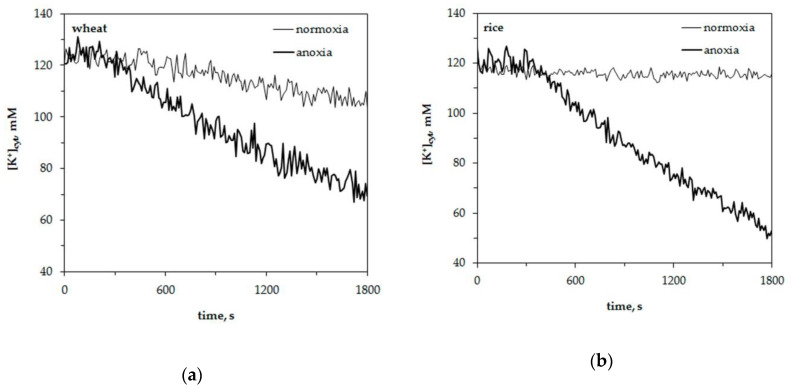
Changes of the free cytosolic K^+^ concentration, [K^+^]_cyt_, in wheat (**a**) and rice (**b**) leaf protoplasts upon imposition of anoxia. Typical single traces in the presence of 10 mM K^+^, 1 mM Ca^2+^ and pH 7.0 in the external medium.

We studied the potassium and proton concentration changes in wheat and rice leaf mesophyll protoplasts under normoxia and anoxia. Wheat was used as it is sensitive to oxygen deficiency and rice as it is resistant to hypoxia. The results of normoxic and anoxic treatment effects on cytosolic potassium concentration, [K^+^]_cyt_, are presented in Figure 1. It shows representative traces of [K^+^]_cyt_ alteration in a single leaf protoplast. The resting level of cytosolic potassium was around 120 ± 5 mM in wheat leaf protoplasts and 125 ± 5 mM in rice mesophyll protoplasts. The resting level of [K^+^]_cyt_ at normoxic conditions had a little decrement within 30 min of measurements of about 7–10 mM in wheat protoplasts and 4–5 mM in rice. Oxygen deprivation led to a more significant drop in K^+^ concentration in both plants. An anoxia-induced decrease in [K^+^]_cyt_ was 1.5 fold in wheat protoplasts (Figure 1a) and twofold in rice protoplasts (Figure 1b). The K^+^ efflux did not start immediately after the imposition of oxygen lack in protoplasts of both experimental plants but after a lag-period. It lasted about 300–400 s in wheat protoplasts and 350–450 s in rice. The duration of the lag-periods for single traces presented in Figure 1 were as follows: 330–350 s in Figure 1a (wheat) and 410–430 s in Figure 1b (rice).

**Figure 2:**
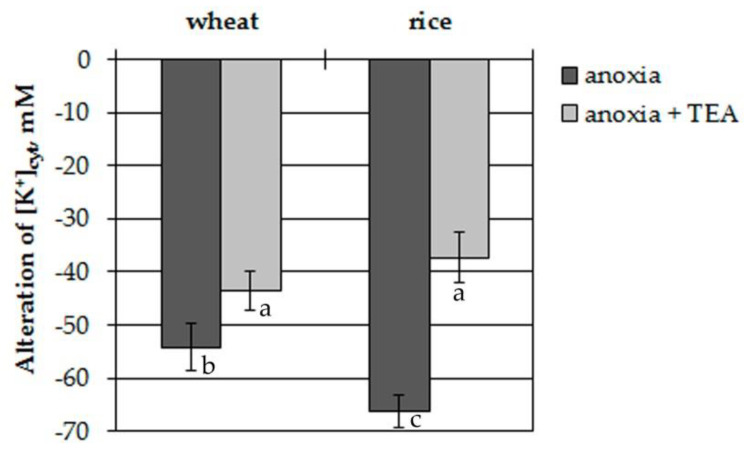
Anoxia-induced alterations of [K^+^]_cyt_ in wheat and rice leaf protoplasts with and without tetraethylammonium (TEA, 1 mM, 30 min pre-incubation). Columns represent mean values ± SD. Values with different letters are significantly different at *p* < 0.05, according to the Least Significant Difference LSD test.

To elucidate the involvement of plasma membrane voltage-gated K^+^ channels in the [K^+^]_cyt_ decrease, the protoplasts were treated with 1 mM tetraethylammonium (TEA). TEA significantly inhibited efflux from the protoplasts of both plants (Figure 2). Inhibition was 20% in wheat protoplasts and 45% in rice ones.

Thus, anoxia led to leakage of K^+^ from the protoplasts of both plants after the lag-period of 300–450 s. The K^+^ efflux was blocked by tetraethylammonium and was higher in rice protoplasts.

### 2.2. Influence of Oxygen Deprivation on pH_cyt_ in Wheat and Rice Leaf Protoplasts 

**Figure 3:**
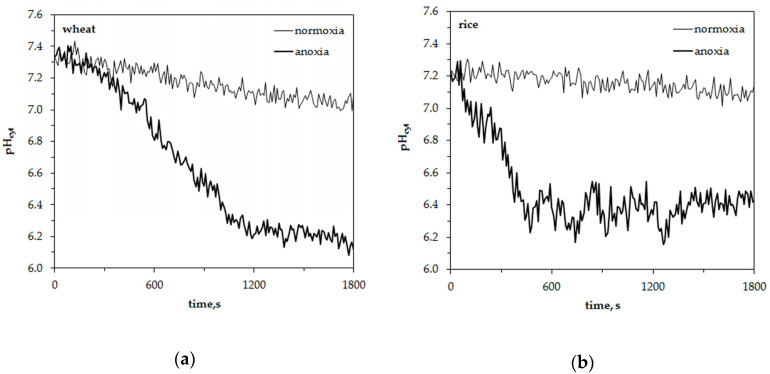
Changes of the free cytosolic H^+^ concentration, pH_cyt_, in wheat (**a**) and rice (**b**) leaf protoplasts upon imposition of anoxia. Typical single traces.

**Figure 4:**
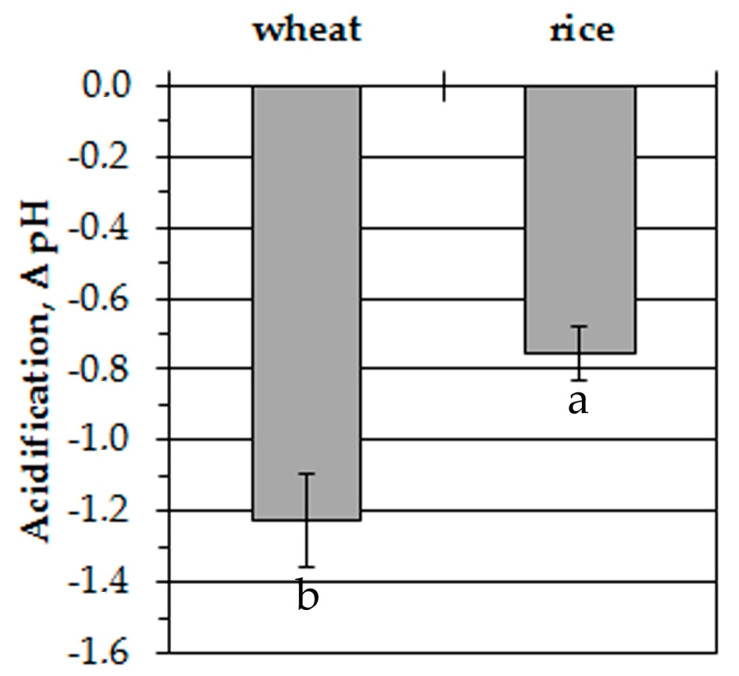
Anoxia-induced alterations of pH_cyt_ in wheat and rice leaf protoplasts. Columns represent mean values ± SD. Values with different letters are significantly different at *p* < 0.05, according to the LSD test.

The representative traces in Figure 3 show anoxia-induced alterations of cytosolic pH in leaf protoplasts of experimental plants. The initial level of pH_cyt_ was somewhat higher in wheat protoplasts (7.3 ± 0.05) than in rice (7.2 ± 0.05). The difference was insignificant. Similar to the measurements of [K^+^]_cyt_, the resting level of pH_cyt_ at normoxic conditions had a little decrement within 30 min of measurements. It was about 0.2 pH units in wheat protoplasts and 0.1 pH units in rice. Anoxic treatment led to a decrease of pH_cyt_ starting after a 200–300 s delay in wheat protoplasts. The pH changed from 7.3 to 6.1–6.2 within 900–1000 s and was kept at a low level until the end of the measurement (Figure 3a). A rapid pH_cyt_ drop in rice protoplasts from 7.2 to 6.2–6.3 occurred during the first 400–500 s of oxygen deprivation (Figure 3b). Followed by several domed oscillations, pH_cyt_ then recovered partially and stabilized at 6.4–6.5 after 1200 s from the beginning of the anoxic treatment. The anoxia-induced pH_cyt_ drop in wheat protoplasts was almost twice as high as in rice (–1.23 and –0.75 units, correspondingly) (Figure 4).

Thus, anoxia caused the acidification of cytoplasm in the protoplasts of both plants but it was more significant and started after the lag-period of 200–300 s in wheat while in rice protoplasts the anoxia-induced decrease of pH_cyt_ was rapid and partially reversible.

### 2.3. Influence of Long-Term Anoxia on Potassium Uptake by Intact Wheat and Rice Seedlings

**Figure 5:**
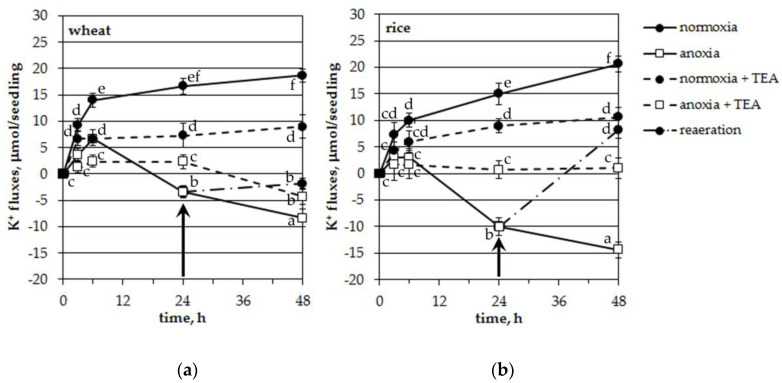
Effects of anoxia and tetraethylammonium (TEA, 0.1 mM) on the net uptake of K^+^ by roots of intact wheat (**a**) and rice (**b**) seedlings. Negative values reflect potassium efflux. Arrows indicate the beginning of reoxygenation treatment (24 h). Mean values ± SD. Values with different letters are significantly different at *p* < 0.05, according to the LSD test.

To detect the total fluxes (influx/efflux) of potassium in/from roots of the studied plants upon anoxia treatment we carried out experiments with intact seedlings. When the roots of the seedlings of both tested plants were placed in an aerated Knop solution (normoxic control), the decrease in K^+^ concentration in the solution, measured by an ion-selective electrode, continued for up to 48 h, reflecting a net uptake of potassium by the roots (Figure 5). The absorption of K^+^ was somewhat higher in wheat in the first 6 h of normoxia. At the end of measurements (48 h), net K^+^ uptake was about the same in both species (18.7 and 20.7 μmol per seedling for wheat and rice, correspondingly). Anoxic treatment arrested the K^+^ influx during 6 h in wheat seedlings and during 3 h in rice. Moreover, after 6 h of oxygen deficiency, K^+^ started to leak from the seedling roots of both plants. Efflux was significantly different from normoxic control and it was significantly higher in rice at 24 and 48 h of anoxia (*p* < 0.05, according to the LSD test).

The use of tetraethylammonium (0.1 mM), a blocker of plasma membrane voltage-gated potassium channels, significantly deteriorated both the acquisition and loss of potassium by seedlings under normoxia and anoxia, correspondingly (Figure 5). TEA inhibition of a net uptake of K^+^ was about 50% in both plants at normoxic conditions. Anoxia-induced K^+^ leakage was completely blocked in rice seedlings while in wheat plants TEA failed to totally inhibit K^+^ efflux after 24 h of anoxia. The TEA-treated anoxic variant showed significantly lesser leakage than the untreated one (Figure 5a). Reestablishment of aeration of the incubation solution after 24 h of anoxic treatment blocked the K^+^ leakage of both plants (Figure 5). Moreover, it even led to a significant reabsorption of K^+^ in rice (Figure 5b).

Thus, anoxia-induced potassium leakage from the roots of intact seedlings was significantly inhibited by TEA. The K^+^ efflux was higher and reversible in rice when compared with wheat seedlings.

### 2.4. Influence of Long-Term Anoxia on the pH of the Incubation Medium of Intact Wheat and Rice Seedlings

**Figure 6:**
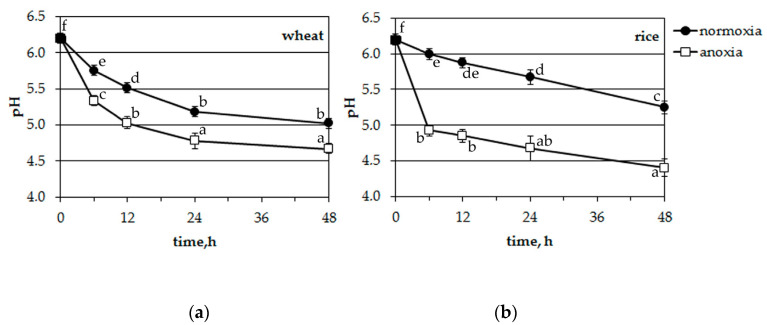
Effects of anoxia on the pH of the incubation medium of wheat (**a**) and rice (**b**) seedlings. Mean values ± SD. Values with different letters are significantly different at *p* < 0.05, according to the LSD test.

Incubation solutions from potassium experiments were also monitored for pH changes (Figure 6). Both plants acidified the incubation medium at normoxic conditions. Wheat seedling roots excreted protons somewhat faster than rice. The anoxic treatment accelerated the acidification of the nutrient solution in both cases (differences were significant at *p* < 0.05, according to the post-hoc LSD test) but in the medium where rice seedlings were growing, acidification was much more rapid (from 6.2 to 4.9 during 6 h of anoxia and from 6.2 to 4.4 during 48 h, Figure 6b).

Thus, anoxia resulted in the acidification of the incubation solution of hydroponically growing plants. Moreover, the acidification of the nutrient solution where rice seedlings were growing was faster and more intensive.

## 3. Discussion

### 3.1. Potassium Changes

The obtained results showed a considerable anoxia-induced decrease of cytosolic potassium concentration, [K^+^]_cyt,_ in the leaf mesophyll protoplasts of both studied plant species but at different lag-periods and the decrease by efflux was more intensive in hypoxia-tolerant rice seedlings than in hypoxia-sensitive wheat (Figure 1). Moreover, the anoxia-induced K^+^ efflux at a cellular level corroborated well with net K^+^ fluxes between the roots of intact seedlings and the external nutrient solution (Figure 5). As the efflux from both species was inhibited by tetraethylammonium, it is likely that K^+^ mainly was transported by K^+^-selective voltage-gated channels. The drop in [K^+^]_cyt_ often originates from K^+^ leakage into the apoplast, although the sequestration of K^+^ ions into the vacuole is also possible during hypoxia [38]. New findings suggest that the potassium efflux might be operated by the activation of GORK channels [25]. The activation depends on depolarization-dependent GORK clustering in the plasma membrane [24]. At normal (unstressed) conditions the activity of GORK is rather limited. It has been suggested for potential dependent channels (including GORK) to form clusters and small-scale rafts that activate channel activity. This process has been shown to be highly regulated by external K^+^ concentration [24].

Reaeration after 24 h of anoxia caused quite different reactions in the two species (Figure 5). It stopped the leakage and resulted in a slight K^+^ reabsorption in the wheat seedlings (about 1.5 μmol per seedling a day) while K^+^ uptake was totally recovered in rice seedlings (18.3 μmol per seedling a day and 15 μmol per seedling a day for the first day of normoxia). This is probably one reason for the anoxia tolerance of rice.

Similar K^+^ leakage during oxygen shortage was reported earlier from the roots of intact wheat seedlings [26], barley [30,39], cucumber [40], roots of rooted cuttings of grape [29] and excised rice coleoptiles [27,28]. The intensity of K^+^ efflux depends on the severity of the oxygen deficiency (hypoxia or anoxia), tissue specificity and age. For example, 1 h of hypoxia caused a twofold decrease of [K^+^]_cyt_ in the cells of the elongation zone in the root epidermis of wild type Arabidopsis but no changes were observed in the mature zone. However, there was a significant increase of [K^+^]_cyt_ in the epidermis of both the elongation and the mature zone under long-term hypoxia (24–72 h) [38]. In barley root, cell hypoxia led to less significant K^+^ fluxes than anoxia [30]. Moreover, cells of the elongation zone of a hypoxia-tolerant cultivar lost more potassium upon short-term anoxic treatment than cells of a sensitive one while it was vice versa in the mature zone of the root. There were no differences in the long-term responses of K^+^ effluxes to anoxia between hypoxia-tolerant and hypoxia-sensitive barley cultivars [30]. On the other hand, hypoxic treatment of the roots of a cultivar of the grape species *Vitis rupestris* sensitive to oxygen shortage resulted in more significant K^+^ leakage when compared with a tolerant *V. riparia* [29]. Furthermore, as in rice, there was a total recovery of K^+^ influx into the roots of *V. riparia* upon the reestablishment of aeration after 20 h of hypoxic treatment while the roots were incapable of K^+^ reabsorption after long-term hypoxia in *V. rupestris*.

Thus, K^+^ efflux is a common reaction developed at cellular/tissue/whole plant levels under hypoxic/anoxic treatment but further investigations are required to reveal the strict specificity between the tolerance to oxygen deprivation and the degree of potassium leakage.

### 3.2. Acidification Caused by Anoxia

The acidification of cytosol is another common reaction in oxygen-deprived cells [14,41]. The imposition of oxygen deficiency is accompanied by a severe decrease in pH_cyt_ in pea, maize [42,43,44], sycamore (*Acer pseudoplatanus*) [45], wheat and rice [46,47]. Our data revealed an anoxia-induced cytosolic acidification in the protoplasts of both plants (Figure 3) but it was more significant in sensitive wheat leaf protoplasts (Figure 4) and the decrease in pH started after the lag-period of 200–300 s (Figure 3a). The rice protoplasts responded to anoxia by a fast decrease of pH_cyt_, which was partially recovered after 500–1200 s of treatment (Figure 3b). Similar dynamics were obtained at a whole plant level; rice seedlings acidified the anoxic solution faster (by 6 h of treatment) than wheat ones (by 12 h). After 12 h the differences between wheat and rice were insignificant (Figure 6). Analogous patterns of pH_cyt_ decrease in wheat and rice root tip cells were reported earlier [46,47].

The acidification of cytosol upon oxygen limitation is due to several reasons. The major reason is low ATP concentration reducing the activity of the plasma membrane proton pumps [43,45,48]. ATP level in the cell is exhausted within 1–2 min after the switch to anaerobic metabolism [49]. Another important H^+^ source is the hydrolysis of ATP and other NTPs [45]. Possible sources of protons are the leakage from the vacuole [50], passive influx from outside [47] and accumulation of organic acid intermediates and products of anaerobic metabolism, predominantly lactate [44,47]. Different patterns of anoxia-induced cytosol acidification may result from different mechanisms in wheat and rice tissues. A partial pH_cyt_ recovery shown in rice protoplasts (Figure 3b) and in root tips [46,47] may have come from the stimulation of ethanol fermentation rather than a lactic one [51]; disposal of lactic acid and the alteration of metabolic pathways can lead to end products other than ethanol or lactate [46,51]. Glycolytic production of ATP provides activity of the plasma membrane [47] and tonoplast proton pumps. A biochemical pH-stat consisting of a shuttle of carboxylating/decarboxylating enzymes is also involved in pH regulation [47,50].

### 3.3. Calcium Involvement in Anoxic Signaling

Both cytosolic [K^+^]_cyt_ decrease and acidification are induced under anoxic treatment. The reactions manifested specificity and appeared due to complicated active and passive ion transport at the plasma membrane. Earlier obtained data highlighted Ca^2+^_cyt_ elevation as another fast oxygen-deficiency triggered reaction at a cellular level [32,34,36,37]. This reaction was detected in different plant species with a wide spectrum of methods [35,52,53]. Using plant cell protoplasts and sensitive fluorescent dyes we revealed clear differences in the timing and amplitude of Ca^2+^ increase for tolerant rice and sensitive wheat. Cells of sensitive wheat were characterized by a lower amplitude and a longer lag-phase of [Ca^2+^]_cyt_ increase compared with rice. Leaf and root protoplasts responded in a similar way to anoxia [35].

### 3.4. A Suggested Model

The obtained results allow us to suggest a general ion signature schedule triggered by anoxia. The events in tolerant rice protoplasts definitely started with cytosol acidification. This reaction was limited to 0.6–0.8 pH units and had very complicated dynamics, probably reflecting the existence of several involved mechanisms. This effect might be linked primarily with the inhibition of H^+^-ATPase due to ATP limitation [43,45,48]. The more rapid reaction in rice seedlings might be linked to fast depolarization of the membrane potential. Within 30–40 s, Ca^2+^ efflux started through potential-dependent plasma membrane channels and further on it accompanied Ca^2+^ release from the intracellular stores [35], predominantly from the mitochondrion due to the dampening of the electron transport chain upon oxygen deprivation [32,34]. The drop in [K^+^]_cyt_ was the last from the tested reactions. It showed about a 10 min lag-period.

Taken together the schedule of primary events of anoxic signal transduction in rice protoplasts could be described as follows. Anoxia-induced inhibition of H^+^-ATPase fast depolarizes the plasma membrane and thus activates Ca^2+^ channels. Cytosol acidification and [Ca^2+^]_cyt_ increases regulate the membrane potential and trigger the complicated mechanism of GORK activation and then provide intensive K^+^ efflux.

In sensitive wheat leaf protoplasts, the reaction was somewhat different. Anoxia application also inhibited H^+^-ATPases but this process had a more prolonged character (lag-period was 200–300 s). On the other hand, the amplitude of the acidification was more pronounced. Changes in the membrane potential also activated the plasma membrane Ca^2+^ channels but its role in cytosol elevation was smaller in comparison with intracellular stores. A lag-period was needed and the reaction was less intensive [35]. Such a delay in [Ca^2+^]_cyt_ shifts explained the lower amplitude of K^+^ efflux in wheat.

The order of primary anoxia-induced reactions still requires additional experimental confirmation. It also desires the insertion of one more significant event such as the elevation of the ROS. In animal cells hypoxia-induced fluxes of Ca^2+^ and K^+^ are performed by oxygen-, ROS- and CO-dependent inward calcium and outward potassium channels [32]. Plants do not possess such channels. All putative analogs in Arabidopsis belong to either the plasma membrane inward K^+^ channels (Shaker type inward rectifier K^+^ channel (AtAKT2) and tandem-pore K^+^ channel (AtKCO4/AtTPK4)) or the Arabidopsis Two-Pore Channel 1 (AtTPC1) in the vacuolar membrane [32]. Nonetheless, anoxia-induced accumulation of free cytosolic Ca^2+^ and pH_cyt_ decreases might directly or indirectly affect potassium transport via AKT2 and AtKCO4 [32] and possibly result in a prolonged lag-period of K^+^ efflux, as revealed in this study.

Potassium efflux was significantly more intensive in rice than in wheat but was reversible after reoxygenation. This fact may be one of the reasons for its tolerance to oxygen deprivation. Taking together, we may conclude that the processes started in anoxia treated cells at a cellular level and then continued to the adaptation of whole plants. Moreover, our results suggest that the dynamic of ion exchange was specific for plants differing in sensitivity to oxygen deprivation.

## 4. Materials and Methods

### 4.1. Plant Material and Growing Conditions

Caryopses of wheat (*Triticum aestivum* L. cv. Kadett, Svalöf Weibull AB, Hammenhög, Sweden) and japonica rice (*Oryza sativa* L., cv. Liman, Rice Research Institute, Krasnodar, Russia) were surface-sterilized for 10 min with 5% NaClO solution, washed several times in distilled water and soaked for 1 h in hot water at 50–60 °C for rice and at 45–50 °C for wheat. The seedlings were grown in beakers on double layers of miracloth (LIC, Stockholm, Sweden) using a continuously aerated Knop nutrient solution [54] (0.2 strength) at an irradiance of 118 W m^–2^ at the top of the shoots and at a 14/12 h light/dark photoperiod, 70% relative humidity and temperatures 22 °C and 28 °C for wheat and rice, respectively, as previously described [35].

### 4.2. Protoplast Isolation and Dye Loading

The isolation of mesophyll protoplasts was carried out by an enzymatic method as described earlier [35]. In brief, the leaves (0.5 g) of 6–7-day-old wheat seedlings were sliced into 0.5–1 mm pieces and treated for 2 h with 1% (*w/v*) cellulase from *Tricoderma resei* (EC 3.2.1.4, lyophilized, 10.0 units mg^–1^ solid, Sigma, St. Louis, MO, USA) and 0.3% (*w/v*) macerase, Maceroenzyme R-10 from *Rhizopus* sp.(EC 3.2.1.4, lyophilized, 0.6 units mg^–1^ solid, Serva, Heidelberg, Germany) solution as described by Edwards et al. [55] with modifications [56,57]. For the isolation of rice mesophyll protoplasts, the concentration of both enzymes was twice higher and the digestion time was 3 h [35]. The protoplasts were purified on a sorbitol gradient [58] and loaded in darkness with potassium- or pH-sensitive fluorescent probes in the form of acetoxy methyl-esters (AM). For measurements of cytosolic potassium concentration, [K^+^]_cyt_, the acetoxy methyl of potassium-binding benzofuran isophthalate (PBFI-AM) was loaded for 3 h at room temperature (20–23 °C) [56,58]. For the monitoring of cytosolic pH, pH_cyt_, the tetra (acetoxy methyl) ester of bis-carboxyethylcarboxyfluorescein (BCECF-AM) was loaded for 1 h at 4 °C [57]. Both ion-sensitive fluorescent dyes were purchased from Molecular Probes^®^ (Eugene, OR, USA). Before measurements, protoplasts were kept in darkness at room temperature for 30 min.

### 4.3. Fluorescence Measurements and In Situ Calibration

The fluorescence intensity ratio was measured with an epifluorescence microscope Axiovert 10 (Zeiss, Oberkochen, Germany) supplied with an electromagnetic filter exchanger (Zeiss), xenon lamp (XBO 75), photometer (Zeiss 01), microprocessor (MSP 201) and a personal computer. Excitation wavelength ratios for the PBFI dye were 340/380 nm and for the BCECF dye 485/436 nm. Emission wavelengths were 500–530 nm for the PBFI and 510–550 nm for the BCECF. All measurements were performed on single protoplasts with a Planneofluar ×40/0.75 objective (Zeiss) for phase contrast. The ratio measurements were performed only with protoplasts of a similar size and properly loaded into the cytosol. Adjustments for signals and noise were made automatically.

For the standard determination of [K^+^]_cyt_, measurements of PBFI-fluorescence were undertaken with protoplasts in separate suspension solutions with concentrations of 0, 10, 20, 50, 80, 100 and 150 mM KCl. NaCl was added to the solutions to give a final concentration of 150 mM [Na^+^ + K^+^] to obtain iso-osmotic conditions [58]. The standard measurements were performed 5–10 min after the addition of 10 μM gramicidin (Sigma, St. Louis, MO, USA) to equilibrate the intracellular and extracellular potassium concentrations. Nigericin (Sigma) was simultaneously added at a final concentration of 5 μM to avoid the pH effect [59].

For pH_cyt_ in situ calibration, the BCECF-AM fluorescence ratio (485/436 nm) corresponding to different pH values was measured on protoplasts in separate standard solutions with pH values ranging from 5.0 to 8.0 with a 0.5 step. The measurements were undertaken 5–10 min after the addition of 5 μM nigericin to equilibrate the intracellular and extracellular pH values [56].

### 4.4. In Situ Anoxic Treatment

The fluorescence measurements were undertaken in a POC-chamber (Bachhofer Lab Equipment, Fisher Scientific, NJ, USA). Micro slides were covered with poly-L-lysine (MW 150,000–300,000, Sigma) at room temperature. Fifty μL of protoplast suspension was placed onto a micro slide and the POC-chamber was mounted above it [35]. The incubation buffer contained 0.5 M sorbitol, 0.05% polyvinylpyrrolidone, 0.2% bovine serum albumin, 5 mM Hepes (Serva), 1 mM CaCl_2_ and 10 mM KCl at pH 7.0. It was degassed for 20–30 min under reduced pressure (–70 kPa) and then saturated for at least 30 min with gaseous nitrogen to an oxygen level less than 25–30 nmol mL^–1^. For the anoxic treatment, protoplasts were submerged by 2 mL of an oxygen-depleted buffer inside the POC-chamber that was immediately closed. For the normoxic treatment, the protoplasts were submersed by the same amount of aerated incubation buffer containing 270–300 nmol mL^–1^ of oxygen. The oxygen concentration in the incubation buffer was detected with an Oxygraph (Hansatech Instruments, Norfolk, UK). To elucidate the effects of tetraethylammonium (TEA, Sigma), which is known to block voltage-dependent K^+^ channels, we pre-treated protoplasts with 1 mM TEACl for 30 min prior to the imposition of anoxia. Measurements were carried out for 30 min (1800 s) and were repeated at least 10 times for each treatment. Figure 1 and Figure 3 demonstrate the representative traces of the specific experiments with single protoplasts from independent cultivations.

The viability of protoplasts was tested using Trypan Blue assay [60]. A sample of the protoplast suspension (50 μL) was mixed with the same volume of 0.4% Trypan Blue (Sigma) solution and was observed under a microscope after 3 min. The percentage of non-stained (alive) protoplasts was calculated from at least seven optical fields on each of four separate slides. The viability of wheat leaf protoplasts was 91.7 ± 3.5% under normoxic conditions and 90.4 ± 3.7% after 30 min of anoxia. The viability of rice protoplasts was 90.5 ± 5.2% and 89.8 ± 5.1% at normoxia and anoxia, respectively.

### 4.5. Measurement of Potassium Uptake by Roots of Intact Seedlings and pH of Incubation Medium

The absorption of K^+^ and the alterations of pH in the incubation medium were tested with 7-day-old seedlings grown as discussed above (4.1.). Prior to the experiments, the caryopses were cut off from the seedlings. Each set of twenty seedlings was placed in glasses containing 20 mL of Knop nutrient solution (0.2 strength). The incubation medium contained 0.636 mM total potassium and had a pH value of 6.2. Glasses were placed into the chamber through which gaseous nitrogen was flushed for 45 min for the creation of anaerobic conditions. The chambers were then hermetically closed and put in the dark in order to prevent the formation of oxygen in the light. Half of the anoxic variants were transferred from anaerobic chambers into normoxic conditions after 24 h of oxygen deprivation and left for 24 h of reaeration. Anaerobic conditions were checked by an Anaerotest^®^ indicator (Merck, Darmstadt, Germany). Control variants were kept in the dark at a normal oxygen level. The concentration of K^+^ was measured with a membrane K^+^-selective electrode EM-101 K (Analit, St. Petersburg, Russia) in the incubation solution after 3, 6, 24 and 48 h of treatment. An EVL-1 M non-polarisable silver chloride electrode was used as a reference electrode. During measurements, the reference electrode was connected to the incubation medium via a U-shaped glass tube 1 mm in diameter and filled with 2% agar in 0.1 M KCl. The electrodes were calibrated with solutions of different concentrations of KCl. The precision of the electrodes was checked by the measurement of the K^+^ level with a flame photometer Flapho-4 (Carl Zeiss, Jena, Germany). For the elucidation of the possible role of voltage-dependent K^+^ channels in total K^+^-fluxes during oxygen depletion, a part of the intact seedlings was treated with 0.1 mM TEA. The blocker was present in the incubation medium throughout the experiment. The shift in K^+^ concentration was calculated in μmol per seedling. The pH of the incubation solution was measured using a Seven Easy S20 pH Meter (Mettler Toledo, Columbus, OH, USA) after 6, 12, 24 and 48 h of treatment.

### 4.6. Statistics

Data in Figure 2, Figure 4, Figure 5 and Figure 6 are presented as mean ± SD for 4–10 experiments. Analysis of variance was done with a GraphPad Prism 5 for Windows. Values with different letters were significantly different at *p* < 0.05 according to the Least Significant Difference (LSD) test. Experiments with protoplasts were carried out in at least four independent cultivations. Fluorescence measurements were made on single protoplasts with at least 10 replicates for each treatment. Experiments with intact seedlings were performed five times.
